# Outbreak of Ampicillin/Piperacillin-Resistant *Klebsiella Pneumoniae* in a Neonatal Intensive Care Unit (NICU): Investigation and Control Measures

**DOI:** 10.3390/ijerph10030808

**Published:** 2013-02-26

**Authors:** Giuliana Fabbri, Manuela Panico, Laura Dallolio, Roberta Suzzi, Matilde Ciccia, Fabrizio Sandri, Patrizia Farruggia

**Affiliations:** 1 Health Care Management, Bologna Local Health Trust, Via Castiglione 29, 40124 Bologna, Italy; E-Mail: giuliana.fabbri@ausl.bologna.it; 2 Hospital Directorate, Bologna Local Health Trust, Maggiore Hospital, Largo Nigrisoli 2, 40100 Bologna, Italy; E-Mail: m.panico@ausl.bologna.it; 3 Department of Biomedical and Neuromotor Sciences, University of Bologna, Via San Giacomo 12, 40126 Bologna, Italy; 4 Hospital Directorate, Bologna Local Health Trust, Bellaria Hospital, Via Altura 3, 40139 Bologna, Italy; E-Mails: r.suzzi@tin.it (R.S.); patrizia.farruggia@ausl.bologna.it (P.F.); 5 Neonatal Intensive Care Unit , Bologna Local Health Trust, Maggiore Hospital, Largo Nigrisoli 2, 40100 Bologna, Italy; E-Mails: matilde.ciccia@ausl.bologna.it (M.C.); f.sandri@ausl.bologna.it (F.S.)

**Keywords:** *Klebsiella pneumonia*, neonatal intensive care units, disease outbreak, infection control

## Abstract

*Klebsiella pneumoniae* is a frequent cause of infectious outbreaks in Neonatal Intensive Care Units (NICUs). The aim of this paper is to describe an outbreak occurred in a 13-bed NICU and the control measures adopted in order to interrupt the chain of transmission. We described the microbiological investigations, the NICU staff compliance to the infection control measures by means of a specifically designed check-list and the control measures adopted. Six cases of primary bloodstream infections sustained by ampicillin/piperacillin-resistant *Klebsiella pneumoniae* were observed over a two-month period. One culture obtained from a 12% saccarose multiple-dose solution allowed the growth of *Klebsiella pneumoniae*. During the inspections performed by the Hospital Infection Control Team, using the check-list for the evaluation of the NICU staff compliance to the infection control measures, several breaches in the infection control policy were identified and control measures were adopted. In our case the definition of a specific check-list led to the adoption of the correct control measures. Further studies would be helpful in order to develop a standard check-list able to identify critical flows in the adhesion to the guidelines. It could be used in different NICUs and allow to obtain reproducible levels of infection control.

## 1. Introduction

*Klebsiella pneumoniae* is a Gram-negative bacillus that belongs to the *Enterobacteriacae* family and is responsible for severe hospital acquired infections (HAIs), including pneumonia and primary bloodstream infections (BSI) [1,2]. It can easily survive in hospitals, reproduce on environmental surfaces and colonize the human skin, bowels, bladder and respiratory tract [3,4]. Transmission is usually from patient to patient via the hands of healthcare personnel [2,5,6].

*Klebsiella pneumoniae* has been identified as one of the most frequent causes of outbreaks reported in neonatal intensive care units (NICUs) [7,8,9,10,11]. In fact it affects immunocompromised subjects as an opportunistic pathogen, and newborns are more prone to the risk of infection due to their immature immune systems, their low weight at birth, and the frequent use of invasive devices and antibiotics [9,12,13].

Four Italian studies showed that most HAIs occurred in newborns admitted to NICUs consisted of sepsis and one of the most commonly isolated microrganisms was *Klebsiella pneumoniae.* They confirmed the importance of low gestational age, low birth weight, invasive devices (mechanical ventilation, umbilical catheterization and CVC), duration of hospital stay and empiric antimicrobial treatment as risk factors for infection in neonates as well [12,13,14,15].

A German study reported that according to the best available scientific evidence nearly 50% of the *Klebsiella pneumoniae* outbreaks occurred in NICUs and were controlled by applying a mix of different infection control measures [11]. More recently the importance of correct antibiotics policies, less use of invasive procedures, hand disinfection/handwashing before and after patient management and isolation precautions have been underlined by several authors [9,16,17].

The aim of this paper is to describe an outbreak which occurred in a NICU and the control measures adopted in order to interrupt the chain of transmission. The development of prevention strategies and procedures is essential in NICUs because of the well demonstrated association between HAIs caused by *Klebsiella pneumoniae* and a significant risk of morbidity and mortality, also due to the increasing *Klebsiella pneumoniae* multidrug resistance [8,9,13,15,18,19].

## 2. Methods

### 2.1. Setting

The outbreak occurred in the 13-bed tertiary level NICU of the Maggiore Hospital, Bologna (Italy). The NICU’s service is staffed by eight paediatricians and 21 nurses and all procedures are performed in an open space. Approximately 250 newborns are admitted to the NICU each year and the healthcare personnel is well trained in the application of HAIs prevention and control measures. Data were collected by the infection control physician.

### 2.2. Case Definition

A case was defined as isolation from blood of *Klebsiella pneumoniae* with signs and symptoms of a BSI (fever (>38 °C), chills or hypotension) according to CDC/NHSN definitions [20]. 

### 2.3. Review of Infection Control Measures/Procedures

When the first four cases of infection were identified a series of measures was implemented. In order to identify potential oversights in the infection control policy, inspections in the NICU were performed daily by the Hospital Infection Control Team, made up of physicians and nurses. They interviewed the NICU staff in order to ascertain any changes in procedures that could have contributed to the outbreak, and evaluated the NICU staff compliance to the infection control measures by means of a specifically designed check-list based on the most recent practical guidelines on HAI’s prevention [21,22,23,24,25] (Table 1). 

**Table 1:** Check-list for the evaluation of the NICU staff compliance to the infection control measures.

Procedure	*Yes*	*No*	*Notes*
Adhesion to hand hygiene guidelines/procedures			
Use of caps in routine activities			
Use of personal protective equipment (caps, mask and scrubs) during invasive procedures			
Respect of asepsis in the management of central venous catheters and venous lines connections			
Disinfection of glucometer after every single use			
Preparation of infusional therapy on a dedicated work surface			
Use of single-patient trays for small medical devices (for example phonendoscope)			
Subministration of a single-dose saccharose solution with a single-patient dummy			
Disposable items in newborn hygiene			
Use of oily solutions in newborn hygiene			
Conjuntival, anal and pharyngeal swabs on admission and every 72 h			
Dipers-scale properly placed (respect of clean pathways)			

The team arranged for the continual surveillance of the patients, the review of all clinical records and a weekly meeting as well. Admissions in the NICU were temporarily suspended in order to help environmental investigations and to prevent further new infections until the identification of the cause. 

### 2.4. Environmental Samples and Microbiological Investigation

Extensive culturing of specimens from the NICU environment, newborn nursery and pharmacy was randomly performed from multidose and monodose solutions, dispensers and corks, TPN pump and keys, feeding bottles, venous lines hubs, working surfaces. The healthcare personnel had hand specimens taken for culturing by using the touch plate method. Isolates were obtained from patients’ conjuntival, anal and pharyngeal swabs on admission and every 72 h. Microbiological samples were inoculated on blood agar and on Herellea agar plates. Bacterial identification was performed by using the semi-automated Vitek2 instrument (bioMérieux, Marcy L’Etoile, France). Antimicrobial susceptibility testing was performed by Vitek2 (AST-GN26 and AST-N089). All isolates of *Klebsiella pneumoniae* that were obtained from patients and from cultures of environmental and hand samples were typed by means of pulsed-field gel electrophoresis techniques.

In order to determine genetic relatedness among strains, repetitive extragenic palindromic PCR (Rep-PCR) using a semiautomated system was applied (DiversiLab; bioMérieux, Marcy L’Etoile, France). The results were confirmed by other two methods home-brew: arbitrary primed polymerase chain reaction (AP-PCR) and random amplification of polymorphic DNA (RAPD-PCR).

## 3. Results and Discussion

Six cases of primary BSI sustained by ampicillin/piperacillin-resistant *Klebsiella pneumoniae* were observed over a two-month period. Baseline characteristics and clinical outcomes of infants were reported in Table 2. The index case had been on the NICU for 10 days at the time of the presentation of the other cases. The birth weights ranged from 820 to 2,340 g and the gestional age from 26 to 36 weeks. Prematurity was the most frequent underlying condition of the infected neonates. All of them had a history of empiric antibiotic therapy with a combination of ampicillin and netilmicin, intravenous catheterization and parenteral nutrition. Only one baby received mechanical ventilation before the isolation of *Klebsiella pneumoniae*.

All six cases were sepsis. The clinical presentation was non-specific and included tachypnea, hypotension, hypothermia and feeding intolerance. C-reactive protein levels were increased in all cases. They all survived and were discharged from NICU.

All the isolates of *Klebsiella pneumoniae* obtained from the patients’ clinical samples had the same antibiogram, showing resistance to ampicillin and piperacillin and susceptibility to cephalosporins, amynoglicosides and imipenem.

Out of 33 environmental specimens only the 10 cultures obtained from NICU yielded bacterial growth; one culture obtained from a 12% saccarose multiple-dose solution used for pain control before invasive procedures allowed the growth of *Klebsiella pneumoniae*; in four further specimens (two from medical devices of a single patient) a coagulase-negative Staphylococcus could be cultured. The hands of 27 out of 29 members of the NICU staff were cultured and failed to yield *Klebsiella pneumoniae*. 644 conjuntival, anal and pharyngeal swabs were cultured.

**Table 2:** Baseline characteristics and clinical outcomes of the 6 infants with Ampicillin/Piperacillin-resistant Klebsiella Infection.

Infant	Gender	Gestational age,	Birth weight,	Major invasive	Type of
		(weeks)	(g)	procedure	infection
A	F	36	2,340	CVC, parenteral nutrition	sepsis
B	M	31	1,220	CVC, parenteral nutrition	sepsis
C	F	26	820	CVC, parenteral nutrition	sepsis
D	F	21	870	CVC, parenteral nutrition	sepsis
E	M	31	1,330	CVC, parenteral nutrition,	sepsis
				mechanical ventilation	
F	M	30	1,770	CVC, parenteral nutrition	sepsis

**Figure 1:**
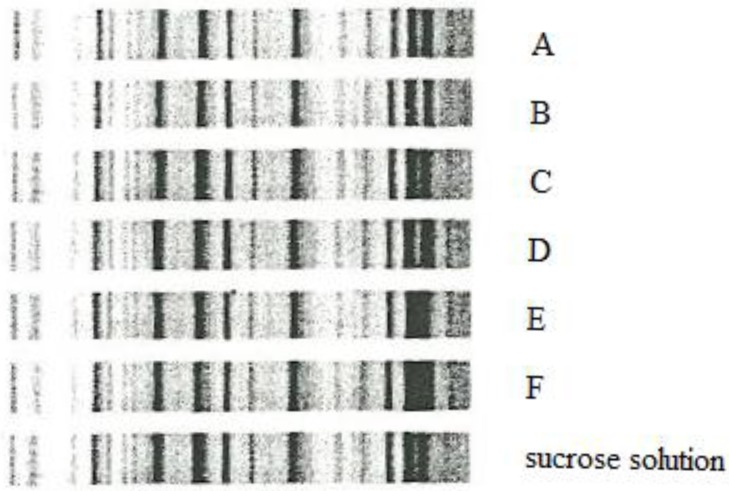
Rep-PCR identified seven isolates with identical banding patterns.

Rep-PCR confirmed that isolates of *Klebsiella pneumoniae* from all six infants and the saccharose solution belonged to the same genotype (Figure 1).

The major risk factors associated with HAIs in NICUs are often difficult to eliminate. Nevertheless multifactorial interventions that improve hygiene and safe procedure have shown to be able to lower the incidence of HAIs [15]. During the inspections performed by the Hospital Infection Control Team in the NICU, using the check-list for the evaluation of the NICU staff compliance to the infection control measures, the following breaches in the infection control policy were identified: the single-dose saccarose solution was administered to several patients, disposable anaesthetic ointment tubes were repeatedly used, the reprocessing of semi-critical multi-use medical devices was incorrect, the preparation of i.v. solutions was not performed in dedicated areas, working surfaces were treated with low-level disinfectant and the use of protection devices was scarce as far as both personnel and parents were concerned. According to the flaws documented in the infection control policy, the following measures were adopted or intensified: specific and timely antibiotic therapy, total respect of the single-dose prescription, personnel and visitors’ hand hygiene and correct use of DPI, hydro-alcoholic solution dispensers installed in every patient box, set-up of a dedicated tray with daily-use medical and hygienic instruments (stethoscope, thermometer, diapers, gauzes, ointments), correct reprocessing of multi-use semi-critical medical devices, implementation of a pathway dedicated to hygienic manoeuvres and garbage disposal from infected infants, set up of clean areas dedicated to aseptic preparations.

## 4. Conclusions

*Klebsiella pneumoniae* is a germ that has proved to be potentially fatal in the NICU [8,9,13,18,19]. According to the well known epidemiology of *Klebsiella pneumoniae*, the outbreak occurred when attention to hygiene measures decreased. In our case we identified a reduced compliance to the general control measures, such as the correct use of DPI or the reprocessing of semicritical medical devices, and to specific procedures.

As it has been stated, establishing hygienic guidelines with an educational program is effective for reducing sepsis rates in NICUs [11]. If compliance to those recommendations decreases the healthcare policy makers should be able to promptly identify the system breaches in order to prevent outbreaks and potentially fatal events. In our case the definition of a specific check-list led to the adoption of the correct control measures in order to limit the outbreak and avoid casualties. Such check-list was based on the recommendations of different guidelines for the prevention of HAIs. Further studies would be helpful in order to develop a standard check-list able to identify critical flows in the adhesion to the available guidelines. Such an instrument could be used in different NICUs and allow to obtain reproducible levels of infection control.
